# Attenuation of LPS-Induced Lung Injury by Benziodarone via Reactive Oxygen Species Reduction

**DOI:** 10.3390/ijms241210035

**Published:** 2023-06-12

**Authors:** Tsutomu Ishihara, Ken-ichiro Tanaka, Ayaka Takafuji, Keita Miura, Tohru Mizushima

**Affiliations:** 1 Department of Chemical Biology and Applied Chemistry, College of Engineering, Nihon University, Fukushima 9638642, Japan; 2 Research Institute of Pharmaceutical Sciences, Faculty of Pharmacy, Musashino University, Nishitokyo 2028585, Japan; 3 LTT Bio-Pharma Co., Ltd., Tokyo 1050022, Japan

**Keywords:** superoxide anions, ARDS, ROS, benziodarone, benzbromarone

## Abstract

As overproduction of reactive oxygen species (ROS) causes various diseases, antioxidants that scavenge ROS, or inhibitors that suppress excessive ROS generation, can be used as therapeutic agents. From a library of approved drugs, we screened compounds that reduced superoxide anions produced by pyocyanin-stimulated leukemia cells and identified benzbromarone. Further investigation of several of its analogues showed that benziodarone possessed the highest activity in reducing superoxide anions without causing cytotoxicity. In contrast, in a cell-free assay, benziodarone induced only a minimal decrease in superoxide anion levels generated by xanthine oxidase. These results suggest that benziodarone is an inhibitor of NADPH oxidases in the plasma membrane but is not a superoxide anion scavenger. We investigated the preventive effect of benziodarone on lipopolysaccharide (LPS)-induced murine lung injury as a model of acute respiratory distress syndrome (ARDS). Intratracheal administration of benziodarone attenuated tissue damage and inflammation via its ROS-reducing activity. These results indicate the potential application of benziodarone as a therapeutic agent against diseases caused by ROS overproduction.

## 1. Introduction

Reactive oxygen species (ROS), including superoxide anions, hydrogen peroxide, and hydroxyl radicals, are generated in various cell types, such as neutrophils, macrophages, fibroblasts, endothelial cells, and vascular smooth muscle cells [1,2,3]. ROS contribute to numerous physiological processes, including host defense, hormone biosynthesis, fertilization, and cellular signaling. In biological systems, enzymes, such as superoxide dismutase (SOD) and glutathione peroxidase, and low-molecular-weight antioxidants, such as glutathione, rapidly remove ROS. However, ROS overproduction is associated with various diseases [1,2,3].

Neutrophils generate ROS in response to stimuli and exhibit bactericidal action. However, activated neutrophils cause excessive ROS production, which contributes to tissue damage and inflammation [4]. ROS overproduction by activated neutrophils has been implicated in the pathogenesis of acute respiratory distress syndrome (ARDS), rheumatoid arthritis, atherosclerosis, and ischemia/reperfusion injury [4,5,6,7]. ARDS is a potentially devastating form of acute lung injury involving neutrophilic inflammation and pulmonary cell death [8,9]. Extensive production of ROS by the infiltrating neutrophils plays an important role in the development of ARDS. Therefore, efforts are underway to develop antioxidants that scavenge ROS or inhibitors that suppress ROS production as therapeutic agents.

Chemically modified SOD derivatives are useful therapeutic antioxidant drugs [10,11,12]. In our previous study, we found that lecithinized SOD (PC-SOD) suppressed lung tissue injury and inflammation by scavenging ROS [10]. In addition, as the major source of superoxide anions in neutrophils is nicotinamide adenine dinucleotide phosphate (NADPH) oxidase (NOX), various NOX inhibitors have been developed [13,14].

However, neutrophils have a short lifespan, and their use in research is limited. HL-60 (a human promyelocytic leukemia cell line) is the most commonly used cell line in neutrophil research. HL-60 cells differentiate into neutrophil-like cells upon treatment with dimethyl sulfoxide (DMSO) or all-trans retinoic acid. In this study, differentiated HL-60 cells were used to evaluate the inhibitory effects of antioxidant candidates on superoxide anion production. In addition, two other cell lines, namely, U937 (a human monocytic leukemia cell line) and KU812 (a human basophilic leukemia cell line) were also used. Although monocytes and basophils are not directly involved in ROS production associated with ARDS progression, these cells, along with neutrophils, express NADPH oxidase 2 (NOX2) in their plasma membrane, and NOX2 is a major source of superoxide anions. Therefore, these cells were also used to validate the results obtained from the experiments performed using HL-60 cells.

In this study, we aimed to analyze a library of compounds to identify one that scavenges superoxide anions or suppresses the production of superoxide anions in leukemia cells. We also investigated the preventive effects exerted by this compound against tissue damage and inflammation in a lipopolysaccharide (LPS)-induced lung injury model of ARDS.

## 2. Results

The HL-60 cells were incubated with each of the approved 1241 drugs from the drug library, and extracellular superoxide anions were chromogenically detected using 2-(4-iodophenyl)-3-(4-nitrophenyl)-5-(2,4-disulfophenyl)-2H-tetrazolium, monosodium salt (WST-1) (see experimental library screening in Supplemental Information; typical results are shown in Table S1). In total, six drugs demonstrated a clear decrease in the absorbance of the solutions. The superoxide anion reducing activity and cytotoxicity of the drugs were further examined in a dose-dependent manner (Table S2 and Figure S1). Finally, benzbromarone was found to exert the highest superoxide anion reducing activity without causing cytotoxicity.

Next, we analyzed specific benzbromarone analogues, namely, benzarone, benziodarone, amiodarone, 2-butyl-3-(4-hydroxybenzoyl)benzofuran (BHBB), and 2-butyl-3-(4-hydroxy-3,5-diiodobenzoyl)benzofuran (BHDB). These analogues contained a benzofuran ring, as shown in Supplemental Figure S2; however, only amiodarone was included in the library. The viability of HL-60 cells incubated with the analogues was examined (Table S3). Amiodarone and BHBB were found to be severely cytotoxic. At 10 μM, amiodarone and BHBB reduced cell viability to 36% and 23%, respectively; however, it reduced to less than 5% for both compounds at 30 μM. In contrast, benzarone, benziodarone, and BHDB were not cytotoxic to the HL-60 cells even at 100 μM. Furthermore, benzbromarone was not cytotoxic at concentrations below 30 μM but was severely cytotoxic at 100 μM (Table S2).

**Figure 1:**
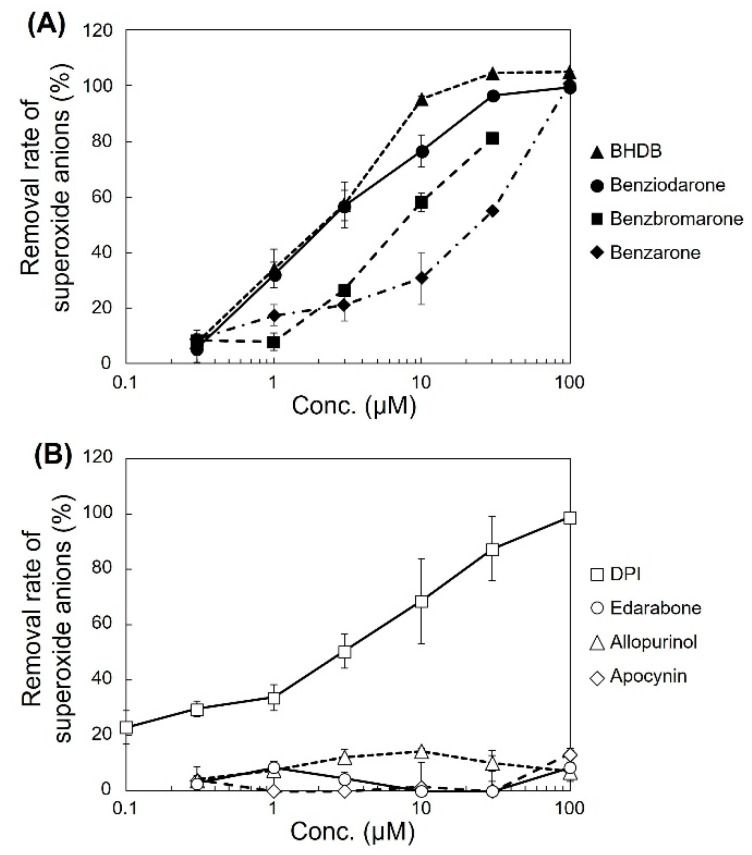
Inhibitory effect of various compounds on extracellular superoxide anions produced by HL-60 cells. Pyocyanin-stimulated HL-60 cells were incubated with various compounds, and superoxide anions in the suspension were colorimetrically analyzed using WST-1. The removal rate of superoxide anions was calculated according to the formula mentioned in the Methods section. (**A**) The compounds benziodarone, benzbromarone, 2-butyl-3-(4-hydroxy-3,5-diiodobenzoyl)benzofuran (BHDB), and benzarone were used. (**B**) The compounds diphenyleneiodonium chloride (DPI), edarabone, allopurinol, and apocynin were used. Each data point represents the mean ± standard deviation (SD) of the values corresponding to three independent wells.

**Figure 2:**
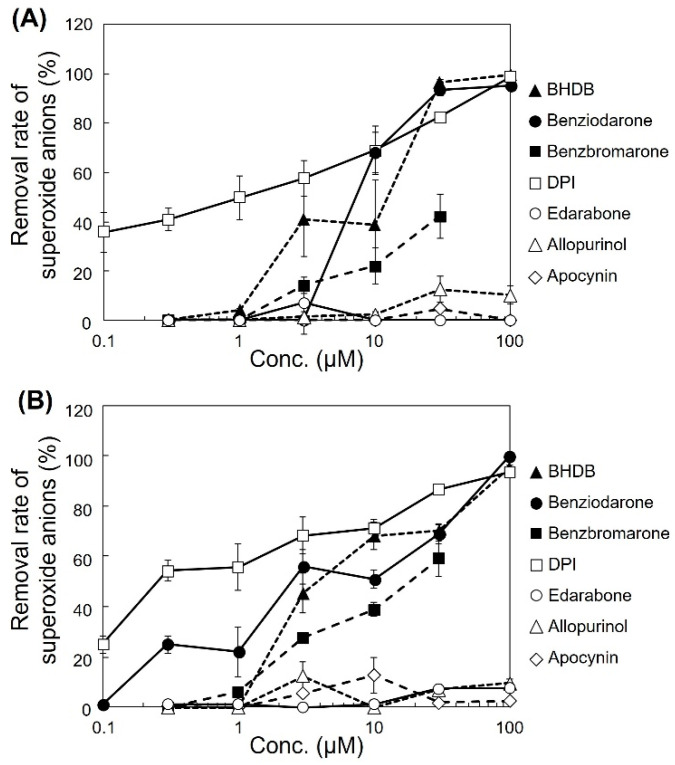
Inhibitory effect of various compounds on extracellular superoxide anions production by leukemia cells. Pyocyanin-stimulated (**A**) U937 cells and (**B**) KU812 cells were incubated with the selected compounds, and superoxide anions in the suspension were colorimetrically analyzed using WST-1. The removal rate of superoxide anions was calculated according to the formula presented in the Methods section. Each data point represents the mean ± SD of the values pertaining to three independent wells.

The ability of the analogues to decrease superoxide anion levels was examined in pyocyanin-stimulated HL-60 cells (Figure 1A). BHDB, benziodarone, and benzbromarone exhibited greater activity than that of benzarone. In particular, BHDB and benziodarone, which differed only in the length of the alkyl chain at position 2, showed the highest activity in reducing superoxide anions. Similarly, four antioxidant candidates, namely, diphenyleneiodonium chloride (DPI), edarabone, allopurinol, and apocynin, were tested, and their activities and mechanisms were compared. DPI showed high activity, whereas edarabone, allopurinol, and apocynin did not (Figure 1B). The inhibitory activities of these compounds in pyocyanin-stimulated U937 cells and KU812 cells showed similar trends as that observed in the HL-60 cells (Figure 2).

**Figure 3:**
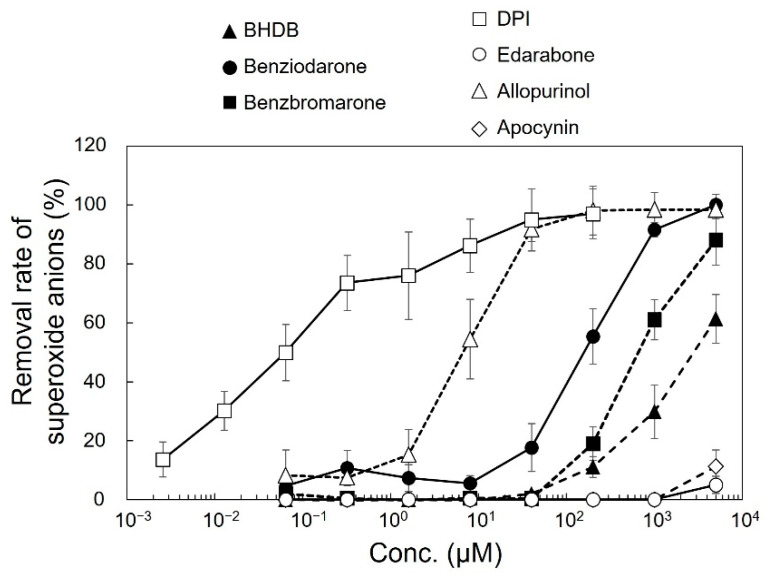
Inhibitory effect of compounds on superoxide anions produced by xanthine oxidase. Superoxide anions were colorimetrically analyzed using the SOD assay kit. The removal rate of superoxide anions was calculated according to the manufacturer’s instructions. The compounds used were benziodarone, benzbromarone, BHDB, DPI, edarabone, allopurinol, and apocynin. Each data point represents the mean ± SD of values pertaining to three independent wells.

The effect of the compounds on the amount of superoxide anions generated by xanthine oxidase was examined using the SOD Assay Kit-WST as a cell-free assay. As shown in Figure 3, superoxide anion levels drastically reduced as the concentration increased for DPI and allopurinol, whereas BHDB, benziodarone, and benzbromarone induced only a small reduction in superoxide anion levels.

**Figure 4:**
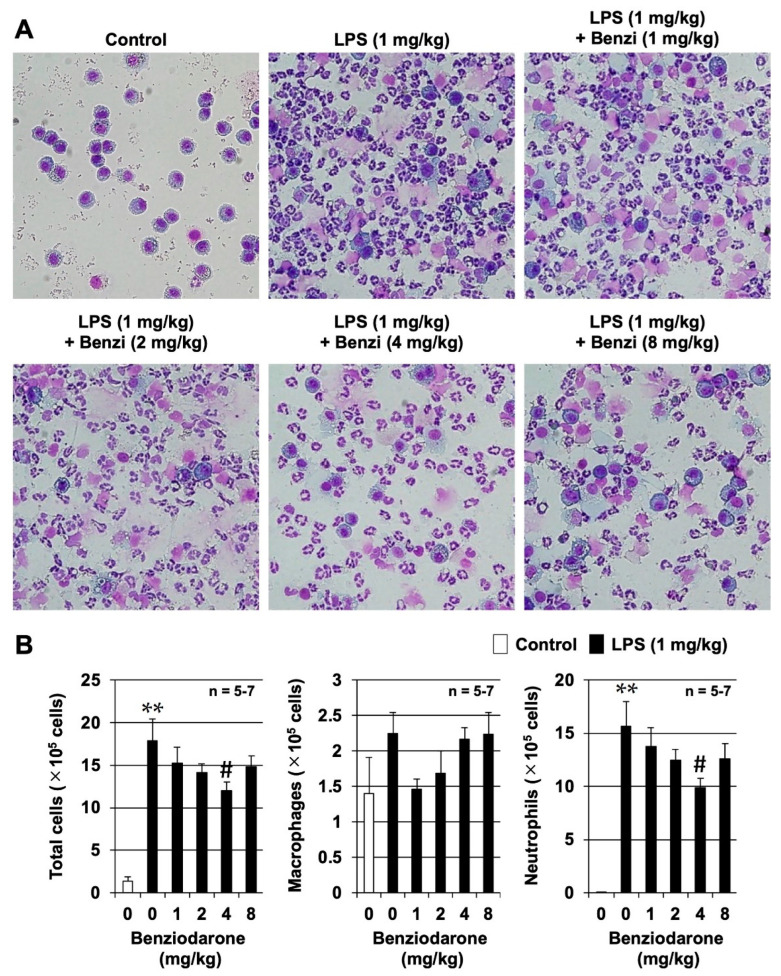
Preventive effects of benziodarone on lipopolysaccharide (LPS)-induced murine lung injury. Male ICR mice were intratracheally administered benziodarone or saline 1 h before LPS administration. Then, the mice were intratracheally administered LPS (1 mg/kg) or saline (Control). Bronchoalveolar lavage fluid (BALF) samples were collected 24 h after LPS administration. (**A**) The BALF cells were stained with Diff-Quik reagents after centrifugation on Cytospin^®^ 4 (Thermo Fisher Scientific, Waltham, MA, USA, Magnification 100×). (**B**) The cell counts for total cells, macrophages, and neutrophils were determined. The values are the mean ± SEM; # *p* < 0.05; ** *p* < 0.01. (**, vs. Control; #, vs. LPS alone).

We further examined the preventive effects of benziodarone at a dose of 1–8 mg/kg body weight on LPS-induced murine lung injury in an animal model of ARDS. Although both BHDB and benziodarone showed the highest activity in reducing superoxide anion concentration in vitro, only benziodarone was selected as the final candidate compound because only benziodarone has been approved and marketed in some countries. We first examined the effect of benziodarone on LPS-induced lung injury by measuring the number of leukocytes in the bronchoalveolar lavage fluid (BALF) 24 h after intratracheal administration of LPS in ICR mice. As shown in Figure 4A,B, the administration of LPS induced a marked increase in the total number of leukocytes, especially that of neutrophils, although little change was observed in the number of macrophages in BALF. In contrast, treatment with benziodarone markedly inhibited the LPS-dependent increase in the total leukocyte and neutrophil counts. In addition, a significant inhibitory effect was observed in the 4 mg/kg benziodarone group (*p* < 0.05, vs. LPS alone). These results suggest that benziodarone administration protects mice against LPS-induced lung injury.

**Figure 5:**
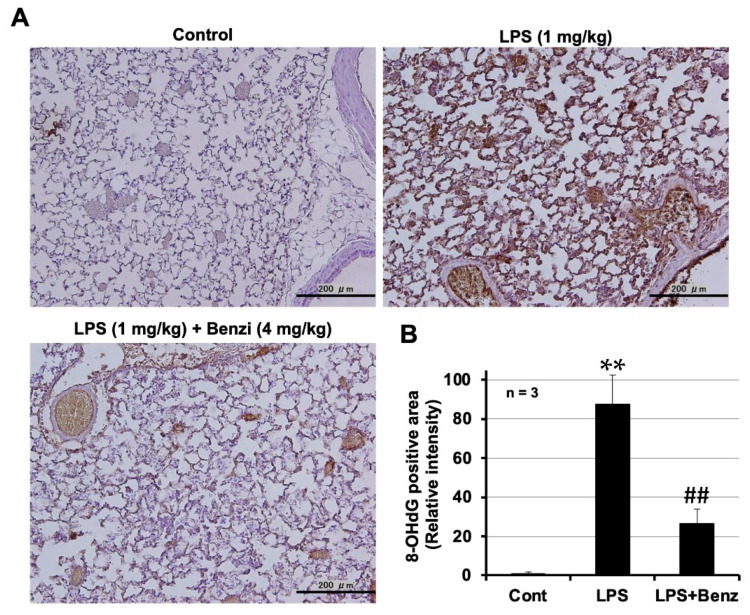
Preventive effect of benziodarone on LPS-dependent increase in reactive oxygen species (ROS) levels. Male ICR mice were intratracheally administered benziodarone or saline 1 h before LPS administration. Then, the mice were intratracheally administered LPS (1 mg/kg) or saline (Control). BALF samples were obtained 24 h after LPS administration. Sections of lung tissue were prepared 24 h after LPS treatment. (**A**) Immunohistochemical analysis was performed; an antibody against 8-OHdG was used (magnification 200×, scale bar = 200 μm). (**B**) The area stained with 8-OHdG was determined using ImageJ software. Values are the mean ± SEM; ** or ## *p* < 0.01. (**, vs. Control; ##, vs. LPS alone).

ROS are involved in the onset and progression of acute lung injury. Therefore, we used 8-OHdG as an oxidative stress marker to monitor the ROS levels. As shown in Figure 5A, a large area of the lung tissue from LPS-treated mice was found to be stained with 8-OHdG, whereas those from mice pretreated with benziodarone did not show this staining. Analysis using ImageJ software showed that benziodarone significantly inhibited the LPS-dependent increase in the 8-OHdG-positive area (*p* < 0.05, vs. LPS alone). These results suggest that benziodarone suppresses LPS-induced lung injury by suppressing the increase in ROS levels.

## 3. Discussion

The two primary approaches for reducing oxidative stress are scavenging ROS with antioxidants and suppressing ROS production with inhibitors; however, which of these approaches is more effective is unclear. In this study, we attempted to identify a compound with the ability to decrease superoxide anions by performing cellular assays using leukemia cells and cell-free assays using xanthine oxidase.

SOD directly catalyzes the dismutation of superoxide anions. In our previous study, SOD effectively inhibited the reductive reaction of WST-1 in both assays [15]. As SOD and superoxide anions cannot cross cell membranes, the results indicated that extracellular superoxide anions could be successfully detected using WST-1 in these assays.

Edarabone, which is an hydroxyl radical scavenger, is used clinically in Japan and the United States to treat ischemia-reperfusion injuries in acute cerebral infarction and amyotrophic lateral sclerosis [16]. Edarabone did not reduce superoxide anions in any of the assays used in this study. This indicates that edarabone is neither a scavenger of superoxide anions nor an inhibitor of superoxide anion production by xanthine oxidase and HL-60 cells, as reported elsewhere [16].

Allopurinol, which is a nonspecific inhibitor of xanthine oxidase [17], suppressed the production of superoxide anions in cell-free assays, but not in cellular assays. This implies that xanthine oxidase does not play a critical role in the production of extracellular superoxide anions in pyocyanin-stimulated HL-60 cells, as the major source of superoxide anions has been reported to activate NOX in neutrophils [18,19].

DPI and apocynin have been studied extensively as NOX inhibitors [20,21]. DPI nonspecifically inhibits various enzymes, including flavoproteins such as the NOX family (catalytic subunits of NADPH oxidases), xanthine oxidase, quinone oxidoreductase, and cytochrome P450 reductase [21,22]. As shown in Figure 1B and Figure 3, DPI effectively reduced superoxide anions in both cellular assays (using HL-60 cells) and cell-free assays. DPI also reduced the production of extracellular superoxide anions in monocytic U937 and basophilic KU812 cells (Figure 2), suggesting that NOX are a major source of extracellular superoxide anions in these cells [23]. Although DPI is useful for studying NOX in vitro, it is not a suitable drug candidate owing to its irreversible binding and off-target effects [21]. In contrast, apocynin did not affect the reduction in the cellular assay, possibly because its activity was much lower than that of DPI [24]. In addition, some reports show that apocynin is a nonspecific inhibitor [25] and not a NOX inhibitor but an antioxidant [26].

Benziodarone, BHDB, and benzbromarone induced little reduction in superoxide anion levels in the cell-free assay but showed a clear reduction in the cellular assay. The IC_50_ values of benziodarone for inhibition of superoxide anions in the cell-free and the cellular assays with HL-60 cells were 180 μM and 2.2 μM, respectively. These results indicated that they have almost no superoxide anion scavenging or inhibitory activity against xanthine oxidase, although benzbromarone has been reported to scavenge superoxide anions [27]. Overall, our results strongly suggest that benziodarone, BHDB, and benzbromarone have strong inhibitory activity against NOX. To date, many studies have reported inhibitors against NOX, but the biggest concern is the lack of selectivity for the specific NOX isoform, NOX2, which is a major source of ROS in neutrophils [13,14,18,19].

**Figure 6:**
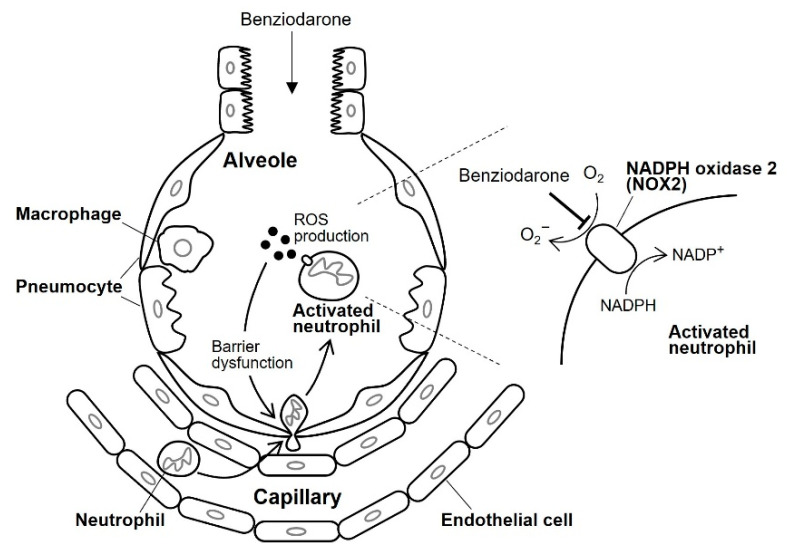
Presumed mechanism underlying the therapeutic effect of benziodarone in an injured alveolus.

Benziodarone and benzbromarone were marketed in the European Union, Japan, and South Africa as oral coronary artery dilators for angina and gout [28,29]. However, they have been withdrawn from the market in several countries due to serious adverse effects, mainly hepatic injuries caused by metabolites reacting with cytochrome P450 enzymes [30]. To overcome this problem, we propose topical application of the drug, which not only reduces the dosage but also prevents the adverse effects associated with systemic administration. ARDS can be a new target disease for these compounds since ROS play a major role in ARDS progression. Furthermore, ARDS can be treated via local administration such as inhalation. In this study, benziodarone, which has the highest inhibitory activity in vitro, was used to evaluate its therapeutic efficacy in mice with LPS-induced lung injury. As shown in Figure 4 and Figure 5, the intratracheal administration of benziodarone attenuated lung injury via ROS-reducing activity. Figure 6 illustrates the presumed mechanism underlying the action of benziodarone on lung injury. Neutrophils migrate into injured alveoli and are subsequently activated to produce several cytotoxic products, including ROS, granular enzymes, neutrophil extracellular traps (NETs), and various pro-inflammatory cytokines [31]. The ROS thus produced cause lung endothelial and epithelial barrier dysfunction, leading to increased neutrophil infiltration into the alveoli. Accumulated neutrophils secrete several cytotoxic substances that promote ARDS progression [31]. We speculate that the intratracheally administered benziodarone was distributed to the alveoli where superoxide anion production by NOX2 was inhibited in activated neutrophils. Although further studies are required to clarify the precise mechanism underlying the inhibitory action, including target molecules and their specificity, these results suggest that benziodarone may be a potential therapeutic drug for ARDS.

## 4. Materials and Methods

### 4.1. Materials

A library of 1241 drugs approved for use in Japan and the United States was supplied by LTT Bio-Pharma (Tokyo, Japan). WST-1 was purchased from Dojindo Laboratories (Kumamoto, Japan). Pyocyanin was purchased from Cayman Chemical Co. (Ann Arbour, MI, USA). Recombinant human CuZn-SOD was obtained from Asahi Kasei Pharma (Tokyo, Japan). LPS from Escherichia coli (055:B5) was purchased from Sigma-Aldrich (St. Louis, MO, USA). In addition, 3-(3,5-dibromo-4-hydroxybenzoyl)-2-ethylbenzofuran (benzbromarone), 2-ehyl-3-(4-hydroxybenzoyl)benzofuran (benzarone), 2-ethyl-3-(4-hydroxy-3,5-diiodobenzoyl)benzofuran (benziodarone), 2-butyl-3-(3,5-diiodo-4-(2-diethylaminoethoxy)benzoyl)benzofuran hydrochloride (amiodarone hydrochloride), BHBB, BHDB, 3-methy-1-phenyl-5-pyrazolone (edarabone), 4-hydroxypyrazolo [3,4-d]pyrimidine (allopurinol), and 4-hydroxy-3-methoxyacetophenone (apocynin) were purchased from Tokyo Chemical Industry (Tokyo, Japan). Diphenyleneiodonium chloride (DPI) was purchased from Enzo Life Sciences (Farmingdale, NY, USA).

### 4.2. Detection of Extracellular Superoxide Anions Generated by Leukemia Cells

Extracellular superoxide anions generated by human leukemia cell lines, including HL-60, U937 DE-4, and KU812 cells, were detected as previously described [32,33]. HL-60 cells and U937 cells were purchased from RIKEN BRC (Saitama, Japan) through the National Bio-Resource Project of the Ministry of Education, Culture, Sports, Science and Technology Japan, and KU812 cells were purchased from the Japanese Collection of Research Bioresources Cell Bank (JCRB Cell Bank, Osaka, Japan). These cells were cultured in RPMI 1640 medium supplemented with 10% fetal bovine serum, streptomycin, and penicillin.

HL-60 cells were pre-cultured in media with DMSO (final concentration, 1.3%) for 4 days to induce neutrophil-like differentiation before the start of the experiment [34]. The cells were then suspended in Hank’s balanced salt solution (HBSS; Nakarai Tesque, Kyoto, Japan) in 96-well plates (7.5 × 10^6^ cells/mL) and incubated with each of the 1241 approved drugs (10 μM) from the drug library for 2 h at 37 °C. Alternatively, benzbromarone, its analogues, and antioxidants (DPI, edarabone, allopurinol, and apocynin) were used instead of the approved drugs. After incubation, pyocyanin and WST-1 were directly added to the wells at concentrations of 50 and 800 μM, respectively. Upon stimulation with pyocyanin, superoxide anions were generated extracellularly by NOX in the plasma membrane. After incubation for 2 h at 37 °C, the absorbance of the solutions was measured at 450 nm (reference wavelength, 630 nm) via spectrophotometry using a microplate reader. The superoxide anion removal rate was calculated using the formula:

$$\mathrm{Removal}\;\mathrm{rate}\;\%=\left\{\frac{\left(\mathrm{OD}\;\mathrm{of}\;\mathrm{control}-\mathrm{OD}\;\mathrm{of}\;\mathrm{blank}\right)-\left(\mathrm{OD}\;\mathrm{of}\;\mathrm{sample}-\mathrm{OD}\;\mathrm{of}\;\mathrm{blank}\right)}{\mathrm{OD}\;\mathrm{of}\;\mathrm{control}-\mathrm{OD}\;\mathrm{of}\;\mathrm{blank}}\right\}\times 100$$

where control refers to cells incubated with pyocyanin and WST-1 (without any drugs); blank refers to cells incubated with WST-1 only (without drugs or pyocyanin), and sample refers to cells incubated with the drugs, pyocyanin, and WST-1.

Compounds that exerted a definitive effect were further investigated at various concentrations. Additionally, we confirmed that the compounds did not directly affect the quenching of the chromogenic tetrazolium salt (WST-1 formazan), as shown in the Supplemental Information.

Alternatively, the cells were incubated with various compounds in HBSS for 2 h at 37 °C, and live cells were counted with a trypan blue dye using a Neubauer cell counting chamber to evaluate the cytotoxicity of compounds.

Furthermore, U937 and KU812 cells that were not treated with DMSO were also used in the same manner described earlier.

### 4.3. Detection of Superoxide Anions Generated from Xanthine Oxidase in a Cell-Free Assay

Superoxide anions were detected using the SOD Assay Kit-WST (Dojindo Laboratories). In this analysis, WST-1 was converted to WST-1 formazan in the presence of superoxide anions generated by xanthine oxidase. After the reaction, the absorbance of the samples was measured at 450 nm (reference wavelength, 630 nm), using a microplate reader. The superoxide anion removal rate was calculated according to the manufacturer’s instructions in the kit.

### 4.4. Therapeutic Effect of the Compound on LPS-Induced Lung Injury in Mice

Male ICR mice (4–6 weeks old) were purchased from Charles River (Yokohama, Japan). The experiments and procedures described here were performed in accordance with the Guide for the Care and Use of Laboratory Animals as adopted and promulgated by the National Institutes of Health and were approved by the Animal Care Committee of Musashino University (protocol code 09-A-2022; date of approval: 17 March 2022).

The mice were anesthetized with isoflurane and intratracheally injected with benziodarone suspended in a 0.45% NaCl solution containing 1% DMSO via the mouth using a P200 micropipette. An hour after benziodarone administration (1–8 mg/kg), the mice were administered a single intratracheal injection of LPS (1 mg/kg) in 0.9% NaCl using a P200 micropipette.

BALF was collected by cannulating the trachea and lavaging the lungs twice with 1 mL of sterile 0.9% NaCl containing 50 U/mL heparin 24 h after benziodarone injection. Approximately 1.8 mL of BALF was routinely recovered from each mouse, and the total number of cells was counted using a hemocytometer. The cells were stained with Diff-Quik reagent (Sysmex, Kobe, Japan) after centrifugation in Cytospin 4 (Thermo Fisher Scientific, Waltham, MA, USA). The ratios of alveolar macrophages and neutrophils to the total cell count were calculated, and the numbers of alveolar macrophages and neutrophils in the BALF were calculated based on these ratios.

Histological analyses were performed as follows. Lung tissue samples were fixed in 10% neutral buffered formalin for 24 h and embedded in paraffin before being cut into 4 μm thick sections. Moreover, 8-hydroxy-2′-deoxyguanosine (8-OHdG), which is one of the major forms of DNA damage induced by ROS, was stained with anti-8-OHdG monoclonal antibody (1:200 dilution) for 12 h. The sections were then incubated with peroxidase-labelled polymer conjugated to goat anti-mouse immunoglobulins for 1 h. Next, 3,3′-diaminobenzidine was applied to the sections, which were then incubated with Mayer’s hematoxylin. The slides were mounted with malinol and visualized under a microscope and digital camera (Olympus DP71, Tokyo, Japan). The 8-OHdG positive area was quantitated using ImageJ software (version 1.39u).

All values are expressed as the mean ± standard error of the mean (SEM). Two-way analysis of variance (ANOVA) followed by Dunnett’s test was used to evaluate the differences between three or more groups. Differences were considered statistically significant at <0.05.

## 5. Conclusions

This study revealed that several benzofuran analogues suppress the generation of superoxide anions in leukemia cells. Benziodarone showed the highest activity in decreasing superoxide anions without cytotoxicity. Although the exact underlying mechanism remains unclear, the results strongly suggest that benziodarone contributes to the inhibition of NOX in the plasma membrane. Intratracheal administration of benziodarone attenuates LPS-induced murine lung injury via ROS-reducing activity. Therefore, benziodarone could be used as a therapeutic agent against diseases caused by ROS overproduction.

## Data Availability

Data can be made available by contacting the corresponding authors.

## Supplementary Materials

The following supporting information can be downloaded at: https://www.mdpi.com/article/10.3390/ijms241210035/s1.
