# Supplementary material for: Attenuation of LPS-Induced Lung Injury by Benziodarone via Reactive Oxygen Species Reduction
Source: Int J Mol Sci. 2023 Jun 12;24(12):10035. doi: 10.3390/ijms241210035 (PMC10298550; doi:10.3390/ijms241210035)
Supplement: Supplementary file 1 [file ijms-24-10035-s001.zip › ijms-2395606-supplementary.pdf]

Supplemental information

Experimental library screening

The first screening

HL-60 cells were pre-cultured in RPMI 1640 medium with dimethyl sulfoxide (DMSO) (final concentration, 1.3%) for 4 days to induce neutrophil-like differentiation before the start of the experiment. The cells were then suspended in Hank's balanced salt solution (HBSS; Nakarai Tesque, Kyoto, Japan) in 96-well plates (7.5×10^6 cells/mL) and incubated with each of the approved 1,241 drugs (10 μM) from the drug library for 2 h at 37 °C. After incubation, pyocyanin and WST-1 were directly added to the wells at concentrations of 50 and 800 μM, respectively. After incubation for 2 h at 37 °C, the absorbance was measured at 450 nm (reference wavelength, 630 nm).

Table S1 shows the results of the analysis of 80 drugs in the library. Four drugs that reduced the absorbance by more than 20% compared with that of the control well were identified. Later, all 1,241 drugs in the library were evaluated in the same manner, and 86 drugs were identified.

Table S1 The first screening performed with 80 drugs from the library

	1	2	3	4	5	6	7	8	9	10
A	0.550	0.496	0.528	0.471	0.536	0.563	0.522	**0.387**	0.602	0.561
B	0.585	0.617	0.584	0.530	0.508	0.523	0.583	0.584	0.566	0.602
C	0.561	0.473	0.536	0.512	0.472	0.516	0.476	0.513	0.567	0.545
D	0.561	0.569	0.532	0.506	0.561	0.557	0.554	0.534	0.520	0.574
E	0.621	0.521	0.547	0.536	0.618	0.581	**0.384**	0.600	0.611	0.603
F	0.563	0.499	0.575	0.596	0.587	0.569	0.567	0.617	0.613	0.578
G	0.541	0.545	0.610	0.559	0.598	0.613	0.530	0.600	0.563	0.572
H	**0.302**	**0.260**	0.554	0.565	0.584	0.529	0.491	0.560	0.518	0.572

The absorbance of the HL-60 cell suspension treated with the drugs, pyocyanin, and WST-1 measured at 450 nm are shown.
The absorbance of the HL-60 cell suspension treated only with pyocyanin and WST-1 measured at 450 nm is 0.553 (control well).
Wells with absorbance reduced by more than 20% compared with that of the control well are shown in red.
Each drug was evaluated in a single well (n=1).

The second screening

Each of the 86 drugs was re-tested in three independent wells using the same protocol described earlier. Alternatively, the cells were incubated with the drugs in HBSS for 2 h at 37 °C and subjected to staining with trypan blue dye. The live cell count was obtained using a Neubauer cell counting chamber to evaluate the cytotoxicity of the drugs. In total, six drugs (toremifene citrate, clomiphene citrate salt, auranofin, benzbromarone, vinorelbine detartrate, and adrenochrome) demonstrated pronounced reduction in the absorbance of the suspension at low cytotoxicity.

The third screening

The superoxide anion concentration reducing activity and cytotoxicity of the six drugs were further examined in a dose-dependent manner. For this analysis the drugs from the library were not used. Instead, the drugs purchased separately were used. Auranofin, toremifene citrate, and clomiphene citrate salt were purchased from Cayman Chemical Co. (Ann Arbor, MI, USA). Benzbromarone was purchased from the Tokyo Chemical Industry (Tokyo, Japan). Adrenochrome was purchased from Sigma-Aldrich (St. Louis, MO, USA). Vinorelbine detartrate was purchased from Fujifilm Wako Pure Chemical Co. (Osaka, Japan). Although toremifene citrate, clomiphene citrate salt, and auranofin did not show apparent cytotoxicity at 10 μM in the second screening, these compounds were cytotoxic above 10 μM in the third screening as shown in Table S2. In contrast, vinorelbine detartrate and adrenochrome were not cytotoxic, but showed less efficacy in reducing superoxide anion levels (Figure S3). Thus, benzbromarone was eventually identified as the compound showing the highest superoxide anion concentration reducing activity while exerting the lowest cytotoxicity among all the compounds screened.

Table S2 Viability of HL-60 cells incubated with each drug

<table>
<tr><th></th><th>0.3μM</th><th>1μM</th><th>3μM</th><th>10μM</th><th>30μM</th><th>100μM</th></tr>
<tr><td>Toremifene citrate</td><td colspan="2">>95%</td><td>73%</td><td colspan="3">＜5%</td></tr>
<tr><td>Clomiphene citrate salt</td><td colspan="2">>95%</td><td>71%</td><td colspan="3">＜5%</td></tr>
<tr><td>Auranofin</td><td colspan="3">>95%</td><td>33%</td><td>10%</td><td>＜5%</td></tr>
<tr><td>Benzbromarone</td><td colspan="5">>95%</td><td>＜5%</td></tr>
<tr><td>Vinorelbine Ditartrate</td><td colspan="6">>95%</td></tr>
<tr><td>Adrenochrome</td><td colspan="6">>95%</td></tr>
</table>

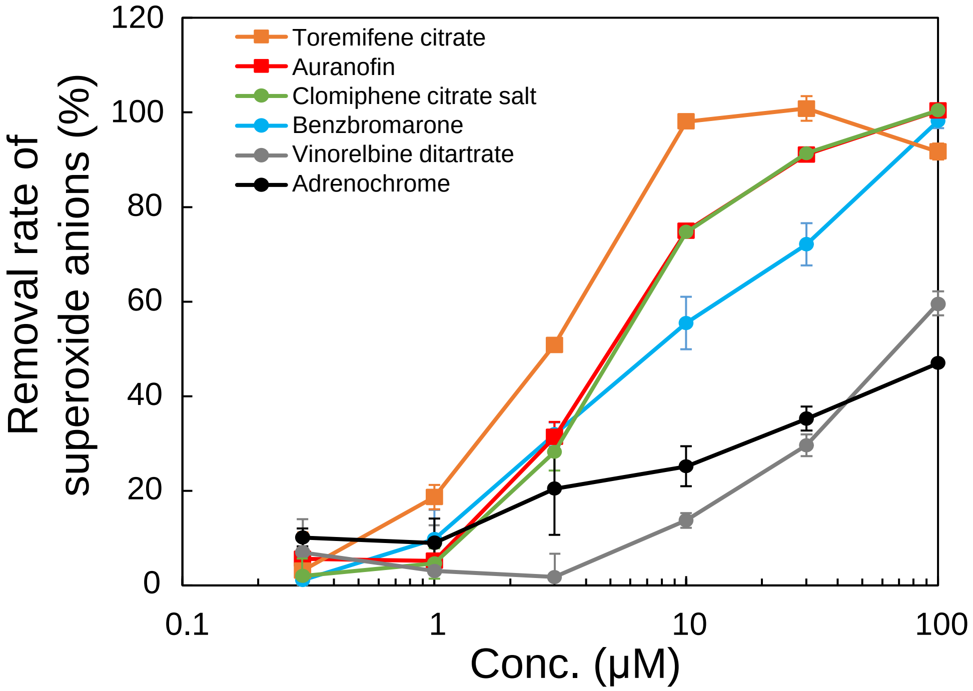

Figure S1 The inhibitory effect of the six drugs on extracellular superoxide anion production in HL-60 cells. HL-60 cells were incubated with each compound, followed by stimulation with pyocyanin. Superoxide anions in the suspension were colorimetrically analyzed using WST-1. The removal rate of superoxide anions was calculated according to the formula presented in the Methods section of the main text. Each data point represents the mean ± standard deviation (SD) of the results pertaining to three independent wells.

Table S3 Viability of HL-60 cells incubated with each compound

<table>
<tr><th></th><th>0.3µM</th><th>1µM</th><th>3µM</th><th>10µM</th><th>30µM</th><th>100µM</th></tr>
<tr><td>Benzarone</td><td colspan="6">>95%</td></tr>
<tr><td>BHBB</td><td colspan="3">>95%</td><td>23%</td><td colspan="2">＜5%</td></tr>
<tr><td>Benziodarone</td><td colspan="6">>95%</td></tr>
<tr><td>BHDB</td><td colspan="6">>95%</td></tr>
<tr><td>Amiodarone</td><td colspan="3">>95%</td><td>36%</td><td colspan="2">＜5%</td></tr>
<tr><td>DPI</td><td colspan="6">>95%</td></tr>
<tr><td>Edarabone</td><td colspan="6">>95%</td></tr>
<tr><td>Allopurinol</td><td colspan="6">>95%</td></tr>
<tr><td>Apocynin</td><td colspan="6">>95%</td></tr>
</table>

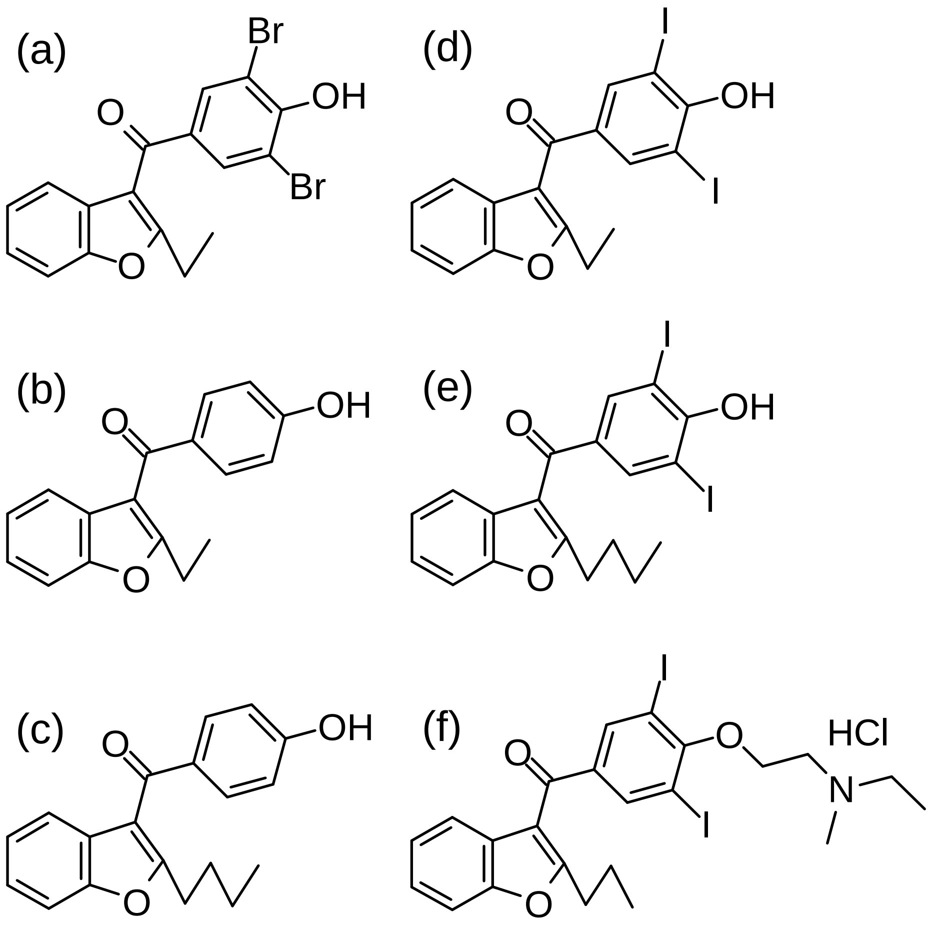

Figure S2 Compounds containing a benzofuran ring.
(a) benzbromarone, (b) benzarone, (c) 2-butyl-3-(4-hydroxybenzoyl)benzofuran (BHBB), (d) benziodarone,
(e) 2-butyl-3-(4-hydroxy-3,5-diiodobenzoyl)benzofuran (BHDB),
(f) amiodarone hydrochloride

Analysis of the interaction between WST-1 formazan and compounds

In this study, superoxide anions were detected using WST-1, which is a cell-impermeable tetrazolium salt. WST-1 is converted to the chromogenic tetrazolium salt (WST-1 formazan) when it reacts with a superoxide anion. Therefore, a decrease in absorbance at 450 nm was considered as a decrease in the amount of superoxide anions. However, some compounds may directly induce quenching of the dyes instead of scavenging superoxide anions.

Therefore, we examined the interaction between WST-1 formazan and the other compounds as follows. A solution of chromogenic WST-1 formazan was collected by incubating pyocyanin-stimulated HL-60 cells with WST-1. The solution was then incubated with the compounds (benziodarone, benzbromarone, BHDB, allopurinol, apocynin, and DPI; 10 μM) for 2 h at 37 °C. No apparent decrease in absorbance was observed for any of the compounds, with a calculated reduction rate of less than 3%. This indicates that the compounds do not directly affect the quenching of the dye.
